# Antidepressant Prescription Behavior Among Primary Care Clinician Providers After an Interprofessional Primary Care Psychiatric Training Program

**DOI:** 10.1007/s10488-023-01290-x

**Published:** 2023-08-20

**Authors:** Shutong Huo, Tim A. Bruckner, Glen L. Xiong, Emma Cooper, Amy Wade, Ariel B. Neikrug, Jane P. Gagliardi, Robert McCarron

**Affiliations:** 1 University of California Irvine, Program in Public Health, Irvine, CA USA; 2 Public Health & Planning, Policy and Design, University of California, Irvine, CA USA; 3 University of California, Davis, Psychiatry and Behavioral Sciences, Sacramento, CA USA; 4 University of California Irvine Department of Psychiatry and Human Behavior, Orange, CA USA; 5 Inland Empire Health Plan, Rancho Cucamonga, CA USA; 6 University of California Irvine School of Medicine, Irvine, CA USA; 7 Psychiatry and Behavioral Sciences, Duke University School of Medicine, Durham, NC USA

**Keywords:** Primary care, Psychiatry, Depression, Antidepressant, Medical education

## Abstract

Primary care providers (PCPs) are increasingly called upon to screen for and treat depression. However, PCPs often lack the training to diagnose and treat depression. We designed an innovative 12-month evidence and mentorship-based primary care psychiatric training program entitled the University of California, Irvine (UCI) School of Medicine Train New Trainers Primary Care Psychiatry (TNT PCP) Fellowship and examined whether this training impacted clinician prescription rates for antidepressants. We retrieved information on 18,844 patients and 192 PCPs from a publicly insured health program in Southern California receiving care between 2017 and 2021. Of the 192 PCPs, 42 received TNT training and 150 did not. We considered a patient as exposed to the provider’s TNT treatment if they received care from a provider after the provider completed the 1-year fellowship. We utilized the number of antidepressant prescriptions per patient, per quarter-year as the dependent variable. Linear regression models controlled for provider characteristics and time trends. Robustness checks included clustering patients by provider identification. After PCPs completed TNT training, “exposed” patients received 0.154 more antidepressant prescriptions per quarter-year relative to expected levels (p < 0.01). Clustering of standard errors by provider characteristics reduced precision of the estimate (p < 0.10) but the direction and magnitude of the results were unchanged. Early results from the UCI TNT PCP Fellowship demonstrate enhanced antidepressant prescription behavior in PCPs who have undergone TNT training. A novel, and relatively low-cost, clinician training program holds the potential to empower PCPs to optimally deliver depression treatment.

## Introduction

Worldwide, depression accounts for the largest fraction of disability-adjusted life years of any mental health condition (“Global, Regional, and National Burden of 12 Mental Disorders in 204 Countries and Territories, 1990–2019,” 2022). In the U.S., major depression ranks as the most common mental health disorder (James et al., 2018, p. 354). In 2020, an estimated 21 million adults in the U.S. had at least one major depressive episode, representing 8.4% of all U.S. adults (Major Depression, 2023). However, the widespread increase in depression in recent years has outpaced a relatively slower growth in treatment rates (Goodwin et al., 2022). Most individuals with depression remain untreated or undertreated (Riolo et al., 2005; Shim et al., 2011; Whiteford et al., 2013). A shortage of psychiatrists contributes to unmet mental health care demand in the U.S. (Bishop et al., 2016; Butryn et al., 2017; Satiani et al., 2018). Whereas the U.S. population has increased by 37% over the last 20 years, the number of psychiatrists has increased by only 12% (Brenner et al., 2017).

One strategy to reduce unmet mental health care needs involves building primary care practices’ capacity to provide frontline mental health services effectively. Given the well-documented scarcity of specialist providers in psychiatry, primary care plays a significant role in the behavioral health delivery system nationwide, particularly in instances where specialty care is unavailable or limited in access (Robinson and Reiter, 2016**).** Primary care clinician providers (PCPs) play a significant role in managing mental illness in that they prescribe more than half of all psychiatric medications (Muench et al., 2022). Additionally, approximately one-third of a PCP’s patients receive treatment for a psychiatric condition (Abed Faghri et al., 2010). Over half of the patients with severe psychological distress obtain mental health treatment from their PCP (Kyanko et al., 2022). Meanwhile, an estimated 60% of mental health care delivery occurs in the primary care setting. Primary care settings also provide lower-cost services and more available access than specialty care (Walker et al., 2015). Regarding the management of depression, the volume of visits for treatment of depression over time has increased significantly to PCPs, along with proportionately less availability and use of psychiatrists (Olfson, 2016).

Clinicians functioning in the primary care setting tend to prioritize physical or general medical needs over mental health-related needs (Poghosyan et al., 2019). Moreover, PCPs often lack the time or training to help patients manage mental health problems in evidence-based ways beyond medication prescriptions (Iskandar et al., 2014; Loeb et al., 2012; Poghosyan et al., 2019; Stilwell et al., 2022). Only around one-half of PCPs have integrated routine depression screening or assessment tools into their practices (LaRocco-Cockburn et al., 2003; Taliaferro et al., 2013). Also, patients treated in primary care practice have higher rates of early antidepressant discontinuation (Croghan et al., 1999). Taken together, available research suggests a need for ongoing educational and collaborative approaches in behavioral healthcare provision to help PCPs.

Even among the large percentage of PCP training programs devoting some time to psychiatric topics, a majority of programs indicate a need for more psychiatric training (Chin et al., 2000; Leigh et al., 2006). Some PCP training programs include no formal or timed clinical education in behavioral health. One recent training project, Project ECHO, shows improvement in behavioral healthcare in primary care settings; however, the program is based on multidisciplinary case reviews using teleconferencing (Hager et al., 2018; Joshi et al., 2022; McBain et al., 2019). Participant feedback includes a desire for more learning time and in-depth lectures from experts (Carlin et al., 2018). As with many medical education initiatives, patient-centered clinical outcomes are lacking.

We designed an innovative and targeted educational initiative to provide psychiatric training to PCPs. The University of California, Irvine (UCI) Train New Trainers Primary Care Psychiatry (TNT PCP) Fellowship is a 12-month longitudinal training program that aims to improve PCPs’ psychiatric clinical knowledge and to enhance PCPs’ confidence in identifying, preventing, and managing psychiatric disorders commonly encountered in the primary care or general medical setting. In a previous evaluation of TNT, PCP trainees at the end of training demonstrate improved attitudes regarding mental health stigma and improved psychiatric clinical knowledge (Neikrug et al., 2022). For instance, in a best-case circumstance, TNT-trained PCPs report that they would feel more comfortable prescribing antidepressant medications to adult patients who screen positive for depression. Prior work, however, does not explore the potential of TNT to affect individual or larger-scale clinical practice patterns.

In this study, we examine whether exposure to a TNT-trained PCP—defined as a PCP after undertaking TNT training—affects the patients’ likelihood of receiving prescriptions for mental health conditions, in this instance, for depression. We focus on antidepressant prescriptions given the reportedly severe undertreatment of depression (Boland et al., 2013; Cassano & Fava, 2002; Thornicroft et al., 2017) We focus on over 18,000 patients in a publicly insured Southern California health program whose medical records are linked to 192 PCPs. We aim for the results of this small study to provide a preliminary indication of the potential of larger rollouts of the TNT program in California may serve as a cost-effective intervention to improve mental health treatment in primary care.

## Methods

### Program Description

The TNT PCP fellowship provides more than 60 h of training outside of traditional clinic work hours in one year from January to December. The majority of the faculty who lead the training are dually trained in primary care areas (internal medicine, pediatrics, or family medicine) and psychiatry. The TNT curriculum includes two 2-day conferences with large-group, interactive learning experiences coupled with small-group discussion to reinforce and incorporate new knowledge. TNT participants further engage in twice-monthly didactics and once-monthly mentorship through video conferencing with TNT faculty. The fellowship provides written materials to supplement learning and mentorship sessions. The curriculum covers a wide range of behavioral health topics and skills relevant to primary care settings, including psychiatric interviewing, mental status examination, and management of psychiatric illnesses (e.g., mood, anxiety, sleep, psychotic, substance misuse, somatic symptom, and personality disorders). Suicide risk assessment, pain medicine/psychiatry, fundamentals of psychopharmacology, cultural formulation, motivational interviewing, and cognitive behavioral therapy are also key parts of the curriculum, emphasizing the importance of empathy and shared humanity as a key to providing effective, efficient patient care. We also invite program graduates to participate in career-long training and mentorship sessions with no additional tuition or cost.

We recruit PCPs to the training program on a rolling annual basis. The total providers who completed the training are 67, 118, and 235 for 2018, 2019, and 2020, respectively.

### Variables and Data

We obtained PCP and patient data from Inland Empire Health Plan (IEHP), a managed care health plan in Southern California, one of the top ten largest health plans in California’s Medicaid Program (Medi-Cal). With a network of over 8,000 healthcare providers, IEHP now serves more than 1.6 million residents in Riverside and San Bernardino counties. These residents represent a racial/ethnically diverse population of Californians who, through means-tested low incomes, qualify for Medi-Cal. Hispanics comprise 47.4% of the total patient population. We identified, from 2017 to 2021, 40 PCPs in IEHP who participated in the TNT training. The 40 PCPs received training in different cohort years (i.e., 10 in 2018, 13 in 2019, and 17 in 2020), which, as we describe below, permits analysis of prescribing behaviors before and after the TNT training. To these 40 PCPs, we additionally selected 152 PCPs who never participated in the TNT training. We selected these “control” PCPs who served patients in similar clinical settings as did the TNT-treated PCPs. This sample resulted in a 3.8 to 1 ratio of control PCPs to TNT PCPs, which lies close to the ratio suggested in the literature (i.e., 4 to 1) above which the gain in statistical power is marginal (Rothman, 1986).

From the clinical records, IEHP provided information on the medication prescription history of 18,844 patients with visits linked to the 192 PCPs from 2017 to 2021. The inclusion of data one year before the start of TNT training, and one year after the training of the most recent cohort, provides greater study power to evaluate antidepressant prescriptions pre- and post-TNT training. Most of these patients visit multiple PCPs, but if they saw any of our identified 192 PCPs *at any point* over the study period, IEHP was able to link their patient record to the PCP. These records include the date (MMYY) of the visit and the medication prescribed.

In the absence of a clinical convention, we used as our key outcome variable the number of antidepressant prescriptions per patient per quarter as an indicator of a PCP’s tendency to screen and treat for depression. We reasoned that an increase in these prescriptions following a PCP’s exposure to the TNT PCP training would reflect the PCP’s improved competency in treating depression. We hypothesized that, in our publicly-insured IEHP population, depression remains under-treated at “baseline” (i.e., before TNT training), consistent with extensive literature (Boland et al., 2013; Cassano & Fava, 2002; Thornicroft et al., 2017). We generated the number of antidepressant prescriptions from the prescription list in each encounter and then aggregated this value to the quarter-year. From the IEHP dataset over a five-year period, we identified 94,274 antidepressant prescriptions.

### Analysis

Our test turns on whether patients seen by TNT-trained PCPs are more likely to receive antidepressant prescriptions. We begin by comparing prescription rates among patients who were seen by a TNT-trained clinician *before* and *after* their start of TNT training. Given that we know the precise timing of the TNT training, we defined a TNT-exposed period as a patient who met with a PCP who was already TNT-trained, and an unexposed period as occurring with a PCP who was not yet TNT-trained. We first compared crude prescription rates among the *same patients*, contrasting the unexposed and exposed periods, and plotted these two rates. The benefit of this unadjusted pre-post comparison involves using a “within-PCP” design to eliminate differences in prescription behaviors across providers as potential confounders.

Next, given secular increases in prescription rates that may correlate with, but are not caused by, exposure to a TNT-trained PCP (which more likely occurs in the second half of the test period 2017–2021), we attempted to minimize this bias in several ways. We applied ordinary least-squares regression techniques and included quarter-year indicator variables as controls (e.g., = 1 if the patient is seen in 1st quarter of 2017, = 0 if seen in other quarters, etc.) as well as a continuous time-trend variable for each of the 20 quarters. In addition, we augmented the patient sample by incorporating 152 additional PCPs—who never received TNT training—and their associated patient cohorts. These patients qualify as never exposed to a PCP-trained clinician but contribute person-time throughout the entire study period, increasing the precision of our regression estimates and helping to account for or control for secular trends in prescription rates. To account for PCP demographic variables that may correlate with prescribing behavior and the decision to receive TNT training, we included gender, specialty, and age of PCP in our regression models.

The above logic led us to estimate a regression equation in which, if the patients receive the prescription after their providers attend the TNT, the time dummy variable equals 1, otherwise, it equals 0. If the patients’ PCP ever participated in the TNT, the dummy variable of TNT training equals 1, otherwise, it equals 0. The interaction term of time and TNT training is our key dependent variable and equals one only among patients receiving care after a PCP received TNT training. We performed data analysis with STATA 16. The literature notes that clustering standard errors (SEs) at greater levels of aggregation (in our case, clustering patients by PCP ID) may appear too conservative in that it could induce a Type II error (i.e., false acceptance of the null) (Abadie et al., 2017). This conservative bias arises when (i) there is likely heterogeneity in treatment effects; (ii) we observe all clusters from a large population of clusters; and (iii) a rather large number of units in each cluster is sampled. Given that these circumstances apply to our test, we estimated all coefficients and SEs, both with and without clustering patient observations by PCP ID, and report both sets of results.

## Results

Table 1 provides sociodemographic and clinical characteristics of the 18,833 patients in IEHP seen by 192 PCPs in our sample. About 5% of patients are homeless. More than half of the patients have a non-low risk of high medical resource burden or more severe illness. TNT-trained PCPs, on average, treat a greater percentage of homeless patients, as well as a higher proportion of patients classified as high clinical risk, than did non-TNT trained PCPs. Hispanic adults comprise nearly half of the IEHP patient population.

**Table 1:** **C**haracteristics of 18,833 patients seen by 40 TNT-trained providers (before and after training) as well as by 152 non-TNT trained providers

	Patients seen by providers *after*TNT training		Patients seen by providers*before*TNT training		Patients seen by non-TNT trained providers	
	N	%	N	%	N	%
Age^a^						
0–20	71	2.08	76	2.82	163	1.28
21–40	1341	39.44	989	36.81	4197	32.93
41–60	1284	37.76	1031	38.37	5162	40.5
61–80	668	19.64	559	20.81	2975	23.34
81+	37	1.09	32	1.19	248	1.95
Homeless	227	6.67	134	4.99	567	4.45
Ethnicity						
Non-Hispanic white	1243	36.56	887	33.01	4273	33.53
Non-Hispanic Black	327	9.6	286	10.64	1222	9.59
Hispanic	1450	42.64	1221	45.46	5552	43.56
Asian	50	1.46	44	1.62	386	3.03
Others	331	9.74	249	9.27	1312	10.29
ACG Risk level^b^						
Low risk	1465	43.09	1083	40.32	5952	46.7
Raising risk	784	23.05	599	22.29	2787	21.87
High risk	1152	33.86	1005	37.4	4006	31.43

b: Adjusted Clinical Groups (ACG) system helps physicians combine a population-level perspective with patient-level behaviors and conditions to manage patients’ risk. High risk means ACG values which represents the higher resource burden or more serious general illness of an individual.

a: Age reflects the current individual age in the insurance system.

**Fig. 1:**
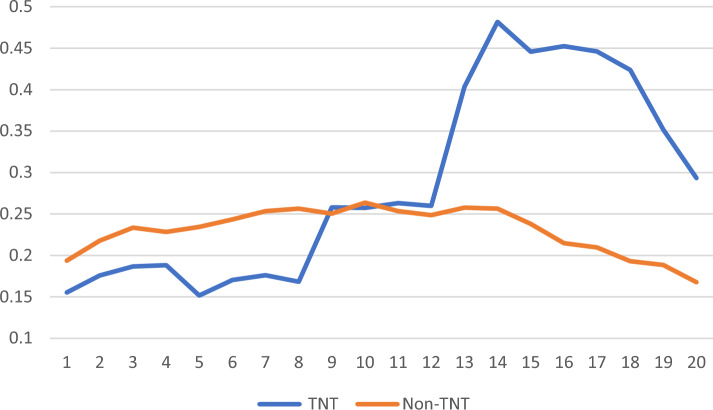
Mean prescriptions per patient per quarter for Antidepressants after the TNT providers attended training versus non-TNT providers from 2017 to 2021. The point 0 is the first quarter of 2017. TNT program started in 2018, so no “TNT provider” appeared in the four quarters of 2017. The average prescriptions per patient per quarter increased after the TNT providers from three cohort (2018, 2019, 2020) attended the training. By contrast, the average prescriptions per patient per quarter of PCPs, who never attended the training, are relatively stable over time

Figure 1 shows that the average antidepressant prescriptions per patient per quarter appears to increase *after* the TNT providers from three cohorts (2018, 2019, 2020) attend the training. By contrast, the antidepressant prescriptions per patient per quarter of PCPs who never attend training are relatively stable over time. The patients of TNT providers receive 0.157 more antidepressant prescriptions per quarter, on average, *after* the providers start their training (relative to the *before* period). In addition, patients of the 192 “never-TNT” providers show qualitatively similar prescription rates to that of patients of TNT providers in the “before TNT” training period (see Appendix Table 3 for details).

Table 2 shows the regression results of the relation between TNT training and antidepressant prescriptions per patient per quarter. Here, the coefficient of interest is the interaction term of the TNT provider and the time period *after* TNT training. Model 1 (left column) represents the base model without clustering SEs by provider; compared to expected levels, the patients of TNT providers, after the TNT training, receive 0.154 more prescriptions of antidepressants quarterly on average (p < 0.0001). Model 2, which controls for provider characteristics, seasonality, and time trends, indicates that PCP age and specialty show associations with the rate of antidepressant prescriptions. Adjustment for these variables, however, does not substantially affect the main results of TNT training (coef: 0.158 more prescriptions of antidepressants quarterly on average [p < 0.0001]). We assessed the sensitivity of antidepressant prescription results to clustering standard error estimates by PCP. Clustering would seem reasonable if PCPs exhibit similar prescribing behavior across their patients, which would render each patient experience (within a particular PCP) as strongly correlated. We, therefore, use the “cluster id” option in STATA, which substantially reduces statistical power but accounts for the nesting of the 18,844 patients within the 192 PCPs in our analysis. As expected, this clustering reduces the precision of the standard error estimates for the interaction term of the TNT provider and the time period *after* TNT training (Model 3, standard error [S.E.] = 0.092 vs. Model 2 S.E. of 0.033). The point estimate, however, remains essentially unchanged and approached conventional levels of statistical detection (coef: 0.158, p = 0.09).

**Table 2:** Coefficients (standard errors) estimating the rate of prescriptions of antidepressants per quarter among patients in the Inland Empire Health Program (n = 18,833) as a function of provider characteristics, time, whether the provider ever received the “Train the Trainer” program, and the interaction of time and provider receipt of the TNT program

Antidepressants	(1)	(2)	(3)
Time	0.003 (0.033)	0.030 (0.033)	0.030 (0.019)
TNT training	− 0.265*** (0.004)	− 0.087*** (0.004)	− 0.087 (0.056)
Time*TNT training	0.154*** (0.033)	0.158*** (0.033)	0.158* (0.092)
Provider Characteristic			
Male gender (ref: female)		0.010 (0.004)	0.010 (0.022)
Specialty			
Family Practice (Ref.)			
General Practice		− 0.143*** (0.076)	− 0.143** (0.072)
Internal Medicine		− 0.010** (0.005)	− 0.010 (0.031)
Nurse		0.144*** (0.007)	0.144** (0.066)
Clinician Assistant		0.331*** (0.012)	0.331*** (0.030)
Age (yrs)			
30–39 (ref.)			
40–49		− 0.013*** (0.004)	− 0.013 (0.022)
50–59		0.109*** (0.006)	0.109*** (0.034)
60–69		0.050*** (0.006)	0.050* (0.028)
70–93		0.053*** (0.011)	0.053 (0.076)
Quarterly trend		0.000 (0.000)	0.000 (0.002)
Seasonality			
Jan.–Mar.(ref.)			
Apr.–Jun.		0.011*** (0.004)	0.011*** (0.003)
Jul.–Sept.		0.007* (0.004)	0.007* (0.004)
Oct.–Dec.		− 0.002 (0.004)	− 0.002 (0.004)
Constant	0.216*** (0.015)	0.212*** (0.005)	0.212*** (0.023)
R^2^	0.004	0.012	0.012

*p < 0.1; **p < 0.05; ***p < 0.01

Model 1 (left column) represents the base model;

Model 2 controls for provider characteristics, seasonality, and time trends.

Both Models 1 and 2 treat all patient observations as statistically independent.

Model 3 was clustered by providers using the “cluster” standard error estimation routine in STATA.

## Discussion

The U.S. and other high-income countries report a shortage of specialty psychiatrists to treat mental disorders. Targeted training of PCPs on screening and treating mental disorders represents one strategy to address the treatment gap. Using data on over 18,000 patients in a publicly insured health plan, we find that patients exposed to TNT-trained PCPs—after the PCPs received their training—show greater rates of antidepressant prescriptions. We note, however, that the precision of results appears sensitive to model choice, owing to the relatively low number of TNT-trained PCPs (i.e., 42) included. Our study, while encouraging, may stimulate replication efforts among a broader set of TNT-trained PCPs to not only increase study power but also to demonstrate generalizability of findings.

Since depression is generally managed by PCPs, who do not push the higher limits of doses observed in clinical trials, real-world doses of antidepressants are usually on the lower end of the dosage spectrum (Sheehan et al., 2008). Our findings suggest that PCP exposure to the TNT program affected PCP prescribing behavior in ways that may have led to more appropriate treatment of psychiatric disorders. Patients in IEHP received more antidepressant prescriptions after their PCPs attended TNT training, which is consistent with other medical training programs focusing on psychiatric education in the primary care setting (Eijk et al., 2001; Henriksson & Isacsson, 2006; Roškar et al., 2010). The result indicates a substantial potential to improve the management of depression in primary care, even though a separate study of a different intervention did not show a difference between PCPs in rates of depression diagnosis or pharmacotherapy after attending the education program (Lin et al., 2001). However, that program consisted of only a 2-hour training. The TNT program, by contrast, provides longitudinal training across an entire year, followed by career-long mentorship, with multiple formats of educational interactions, including faculty mentorship groups, which may contribute to program effectiveness.

Limitations of our study include the relatively few TNT-trained PCPs included in our regional analysis. This circumstance limited statistical power when clustering patient observations by PCP (see Model 3 of Table 2). We, however, note that the literature describes the overly conservative nature of SE estimation (i.e., inflation of SEs and risk of false acceptance of the null) when clustering observations in cases such as ours (Abadie et al., 2017). For this reason, we encourage the reader to interpret all sets of results (i.e., those from Fig. 1and from columns 2 and 3 in Table 2) in light of the relatively few TNT-PCPs available in the IEHP dataset.

We intend for future work to link TNT-trained PCP data to patient information across a broader geography in California than for Riverside and San Bernadino counties alone. Whereas we have no reason to believe that these counties are unique in terms of PCP prescribing behavior, we await larger-scale studies to determine generalizability. Another limitation involves the unknown accuracy of using Medi-Cal claims data for PCP diagnoses of mental health disorders, as claims data are used primarily for billing and reimbursement. Nevertheless, the focus of claims data on billing and reimbursement strengthens the measurement validity on the medication prescription data given that this information has reimbursement implications.

In addition, we have no information about the enrollment periods for each patient. The duration of enrollment can influence the patients’ exposure to depression care and subsequently affect prescribing rates. Variations in enrollment volume over time, however, do not likely bias our analysis because we rigorously control for secular time trends in prescribing rates**.** Finally, we cannot rule out the possibility that PCPs who choose to undertake TNT training fundamentally differ from non-TNT trained PCPs in terms of willingness and motivation to treat persons with psychiatric disorders. We believe the non-random selection into TNT training is unlikely to drive results in light of the unadjusted analysis of “within PCP” prescription rates before and after the TNT training—which examines only TNT-trained providers—showing similar results to the adjusted regression analyses with a larger set of PCP controls.

The next steps in our inquiry will focus more on the clinical effects of increased antidepressants prescription and the quality of the management of mental health in primary care settings. Studies find that the greater prescriptions of antidepressants are associated with fewer suicides (Haukka et al., 2009; Henriksson & Isacsson, 2006; Valuck et al., 2009). Some research also shows that educating PCPs can help protect against patient suicide, which suggests a potential benefit of PCP training on this outcome (Rihmer et al., 2001; Szanto et al., 2007). In addition, a high rate of early antidepressant discontinuation occurs in primary care settings (Dunn et al., 1999; Simon et al., 1993). The discontinuation of antidepressant treatment for depression beyond the first months can increase the risk of relapse (Bambauer et al., 2007; Geddes et al., 2003; “Practice Guideline for the Treatment of Patients with Major Depressive Disorder (Revision). American Psychiatric Association,” 2000). We will therefore examine whether training from TNT program improves the continuity of antidepressant treatment.

Our findings, if replicated on a larger scale, may hold implications for practice and post-graduate and continuing medical education. The UCI TNT PCP Fellowship is designed and implemented by dual-boarded clinicians in primary care psychiatry taking into account the data regarding components of successful programs, including continued educational interactions, a continuous relationship between teacher and learner, interactive participation, and clinical relevance (Gilbody et al., 2003; Hodges et al., 2001, pp. 1950–2000). The TNT PCP Fellowship involves training in prevention, assessment, and provision of psychiatric care within the clinical structure and acknowledging the competing demands on PCPs. TNT PCP training holds potential to be scaled to a larger set of PCPs and may assist with bridging the well-documented psychiatric treatment gap, which is especially large among publicly-insured patients.

## Appendix

**Table 3:** Prescriptions per patient per quarter on average before and after the TNT provider attending the training vs. Non-TNT providers, for each of three cohort years 2018, 2019, and 2020, by characteristics of providers

	2018			2019			2020		
	After TNT	Before TNT	Non-TNT	After TNT	Before TNT	Non-TNT	After TNT	Before TNT	Non-TNT
Total	0.236	0.123	0.219	0.336	0.269	0.233	0.522	0.185	0.241
Gender									
Male	0.241	0.097	0.266	0.303	0.302	0.268	0.538	0.167	0.267
Female	0.213	0.238	0.184	0.382	0.223	0.208	0.514	0.193	0.220
Specialty									
Family Practice	0.359	0.136	0.216	0.323	0.284	0.235	0.216	0.211	0.246
General Practice	0.197	0.094	0.257	.	.	0.245	.	.	0.235
Internal Medicine	.	.	0.191	0.308	0.093	0.210	0.218	0.229	0.215
Nurse	0.129	0.335	.	0.567	0.222	.	0.946	0.166	.
Clinician Assistant	.	.	.	.	.	.	1.302	0.039	.
Age									
30-39	0.381	0.124	0.122	0.349	0.161	0.164	0.559	0.157	0.203
40-49	0.127	0.345	0.141	0.331	0.317	0.161	0.185	0.201	0.181
50-59	0.194	0.053	0.311	0.425	0.288	0.313	1.031	0.163	0.298
60-69	0.233	0.218	0.317	0.316	0.242	0.318	0.233	0.234	0.308
70-93	0.209	0.248	0.143	.	.	0.147	.	.	0.148
